# Short-term effects of low-level laser versus ultrasound therapy on children’s neck posture after long-term use of electronic devices

**DOI:** 10.1007/s10103-023-03895-y

**Published:** 2023-10-13

**Authors:** Ghadeer Mohammed Rabie, Kamal Elsayed Shokry, Jehan Alsharnoubi

**Affiliations:** 1 Cairo, Egypt; 2 Department of Pediatric Physical Therapy Faculty of Physical Therapy, Cairo University, Cairo, Egypt; 3 Department of Pediatrics, National Institute of Laser Enhanced Sciences (NILES), Cairo University, Giza, Egypt

**Keywords:** LLLT, VAS, Cervical straightening, Children

## Abstract

The purpose of the study was to compare the effect of low-level laser therapy (LLLT), ultrasound therapy (US), and strengthening and stretching exercise in children diagnosed with cervical straightening caused by long-term use of electronic devices. A total of 60 patients from 12 to 18 years of both gender diagnosed with straight neck syndrome which is losing the normal C shape of cervical vertebrae. Patients were randomized into three groups. In group A, patients received LLLT plus exercise, while in group B, they received US plus exercise, and group C received exercise. In group A and B, Cobb angle and VAS showed a significant improvement (more significant in group A) with *P* < 0.001, while in group C *P* = 0.006. LLLT, US, and exercise improved the cervical straight curve, and reduced pain with maximum effect was done by LLLT.

## Introduction

Due to the proliferation of high-technology devices in recent years, electronic media have become deeply embedded in modern culture. Studies suggest that children spend a significant amount of their waking hours each day using their screens [1].

Research measured the degree of lateral neck flexion by calculating the angles between the acromion process, C7, and the base of the skull. No matter which side the neck is flexed, persistent lateral neck flexion will cause ligament instability and chronic neck pain. Not only will prolonged neck flexion cause pain, but it will also have a negative impact on proprioception and neck sensation. Dizziness, disorientation, and nausea may be felt as a result of the reduction in proprioception and feeling.

The assessment of pelvic obliquity during smartphone use was discovered in the same investigation. After using a smartphone, the pelvic obliquity measured from T12 to the posterior superior iliac spines on both the left and right exhibited statistically significant alterations. Because the spinal column is a closed kinematic chain, pelvic obliquity may produce pain in the low back and upper thoracic area [2].

Straight neck deformity was recently used to describe a group of musculoskeletal symptoms, including neck and shoulder pain, that are associated with improper and extended neck posture while texting during the day. The continuous head-down tilt posture has been suggested to stress muscle activity, creating changes in the cervical spine and also indicating that greater head flexion may be associated with increased muscle loads and strain on the neck region [3].

The spine should be neither excessively curved nor straight, but within a range of normality. Maintaining an unfavorable posture can lead to postural syndromes including excessive thoracic kyphosis and a protruding chin, which can lead to insufficient muscular contractions, postural muscle weakness, and tiredness. Reduced activity in the postural musculature can lead to overuse of the mobilizer muscles, resulting in discomfort, stiffness, and discomfort. People with protruding chin postures who utilize their smartphones for extended periods of time have been observed to experience pain as well as fatigue as a result of smartphone usage [4, 5].

Straight neck syndrome is a common musculoskeletal condition with the absence of natural curvature along the upper cervical spine. It is characterized by neck pain, postural changes, lower back and/or shoulder pain, and general concentration issues. The normal C-shaped lordotic curve is seen from the side. The curve of the cervical spine is measured using a variety of procedures, the most frequent of which is the measuring of Cobb angle, which defines angles among 20° and 60° are considered normal cervical curvature [6].

VASs are well-known as reliable measurement methods for quantifying pain intensity and assessing psychological states affected by pain [7].

Low-level laser therapy (LLLT) provides bio-stimulation, just enough to induce a response in body tissue and cell function [8]. Ultrasound (US) applies mechanical as well as thermal effects by enhancing blood flow and metabolic activity. In addition to heat, non-thermal mechanisms involving ultrasonic cavitation as well as mechanical stress also contribute to US effects [9].

The natural “C”-shaped cervical lordosis determines how much or how little the cervical spine can move in all directions. The cervical spine’s C5 to C7 intervertebral joints, together with the atlanto-occipital and atlantoaxial joints, are all involved in each of the six movements. Any reduction in cervical joint range of motion would affect the sum and combination of all movements at the cervical spine. Flexion and extension were reduced in cases of straightened cervical lordosis due to the cervical spine’s alignment. When the spine is in lordosis, it is already stretched out and in a position where extension motion is restricted. If extension motion is restricted, flexion naturally decreases, which then affects all other conceivable movements, including lateral flexion and rotations. In essence, exercises with resistance essentially make the cervical extensors stronger, allowing them to regain and keep the cervical lordosis in place, which in turn allows for flexible motion at the cervical intervertebral joints. A straightened cervical lordosis maintains the cervical spinal extensors stretched all the time, which over time weakens them and puts more pressure on the anterior disc, which in turn strains the posterior longitudinal ligaments and creates pain. [10].

## Aim of work

The purpose of this study is to evaluate the effect of low-level laser therapy, therapeutic ultrasound, and strengthening and stretching exercises for straightening of cervical spine curve deformity in children after long-term use of electronic devices specifically smart phones.

## Patient and method

The study was a prospective, randomized, controlled study. Sixty children from 12 to 18 years old of both sexes were recruited by envelope method from the pediatric laser clinics at the National Institute of Laser Enhanced Sciences in Cairo University (NILES) and the pediatric physical therapy clinic at the Faculty of Physical Therapy, Cairo University. It was approved by the ethical committee.

Each participant and his parent was informed about the nature, aim, and benefits of the study and signed a consent or his gradient according to declaration of Helsinki. The patients were randomized into three equal groups by choosing from a closed envelope. The patient selects one of 60 closed envelops written in side it the method of treatment whether laser or ultrasound therapy or physiotherapy.

### Inclusion criteria

Patient will be included in the study if they have spent long time using electronic devices (such as mobile phones, tablet, laptop, or television) for more than 6 months.

Patient diagnosed with straight neck syndrome.

Patient aged from 12 to 18 years old from both sexes.

### Exclusion criteria

Cervical congenital anomalies: simple fused vertebrae due to segmentation defect or complex craniocervical instability as unfused clivus (UC), abnormal facet complex, and accessory ossicles, ponticulus posticus, and arch anomalies [11]

Chronic deformity more than 5 years.

Cervical fracture with nail fixation.

Sixty children aged from 12 to 18 years old were distributed into three groups of equal number:

Group A: 20 patients (except 1 patient did not complete the study) received low-level laser therapy three sessions each week for 4 weeks.

Group B: 20 patients received therapeutic ultrasound therapy, three sessions each for 4 weeks.

Group C: 20 patient received stretch and strength exercise three sessions each for 4 weeks.

All groups had stretch and strength exercise during treatment as follows:

**Stretching the neck flexors**: Place one hand at the base of the person’s head, and the other hand should be on top of the head.

The head was gently moved back.

**Neck extensor stretch**: The patient was sitting straight up in the chair, while this stretch was being performed on the suboccipital and long extensor muscles. Then, while the patient was still gazing straight ahead, the chin was softly moved back. With one hand holding the patient’s chin back, the other hand gently dragged the patient’s top of the head forward.

**Sternocleidomastoid**: Patients instructed to sit up straight with back straight and chest up. The patient had one hand on their chin and the other on the top of their head. The neck flexed to the other side as the head rotated to one side. The head then turned and extended back while retaining side flexion and rotation.

*Strengthening exercise for neck flexion, extension, side bending, and lateral rotation.*

The patient was perched on a chair, and one hand’s palm was pushed against the patients forehead. The exercise was then performed while maintaining pressure on the patient’s head’s side to prevent neck bending. The exercise performed for five times. The activity then shifted to the opposite side.

### Devices used

#### Low-level laser

**Fig. 1:**
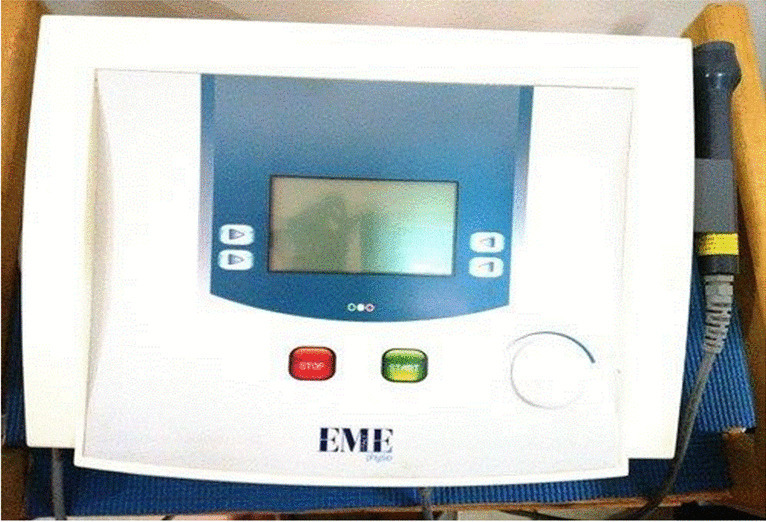
Low-level laser used in our study

**Fig. 2:**
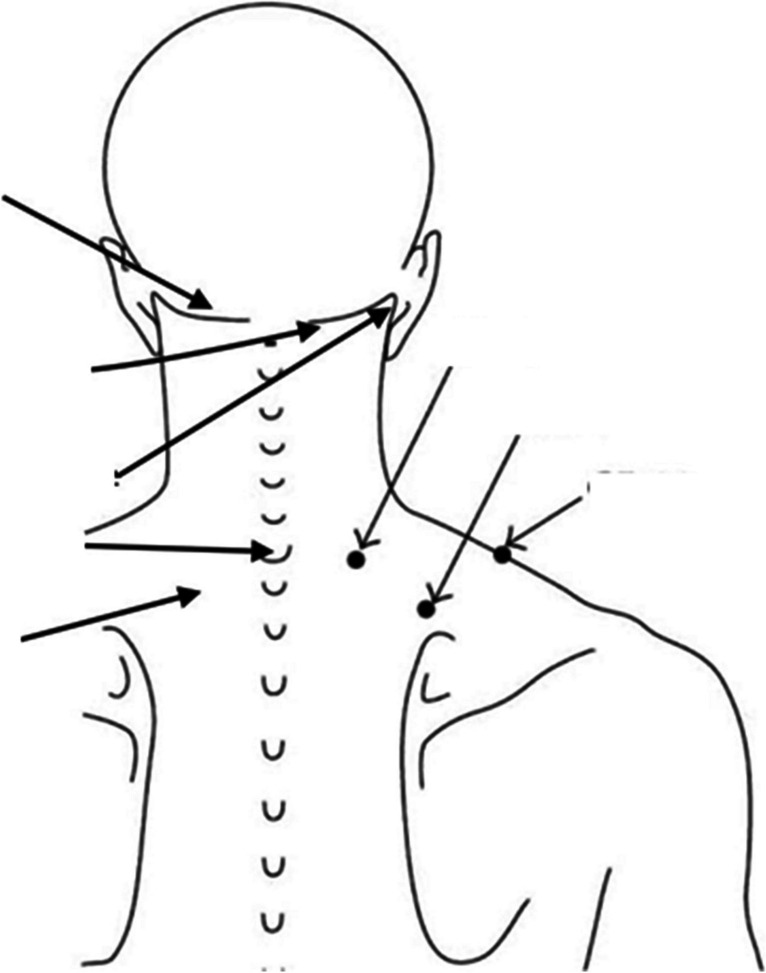
Diagram showing the points where both devices were used

Group A got LLLT with low-level diode laser 810-nm (invisible infrared) laser light generating 500 mw output, laser probe with 5 diodes of 100 mw each (500 mw in total) + guide light (EME brand, model; LIS 1050, 905 length of diode emission) as shown in Fig. 1, using duty cycle 50%, intensity 100%, power density 12 mW/cm^2^. The laser “probe” head placed directly upon points of the cervical spine with the subjects head in neutral position. Each point treated for 2 mins, 8 points were marked across Trapezius and sternocleidomastoid muscles bilaterally to cover 24 cm^2^ for total time 18 mints, with 2 J/cm^2^ at every point as shown in Fig. 2 [12].

#### Therapeutic ultrasound therapy

**Fig. 3:**
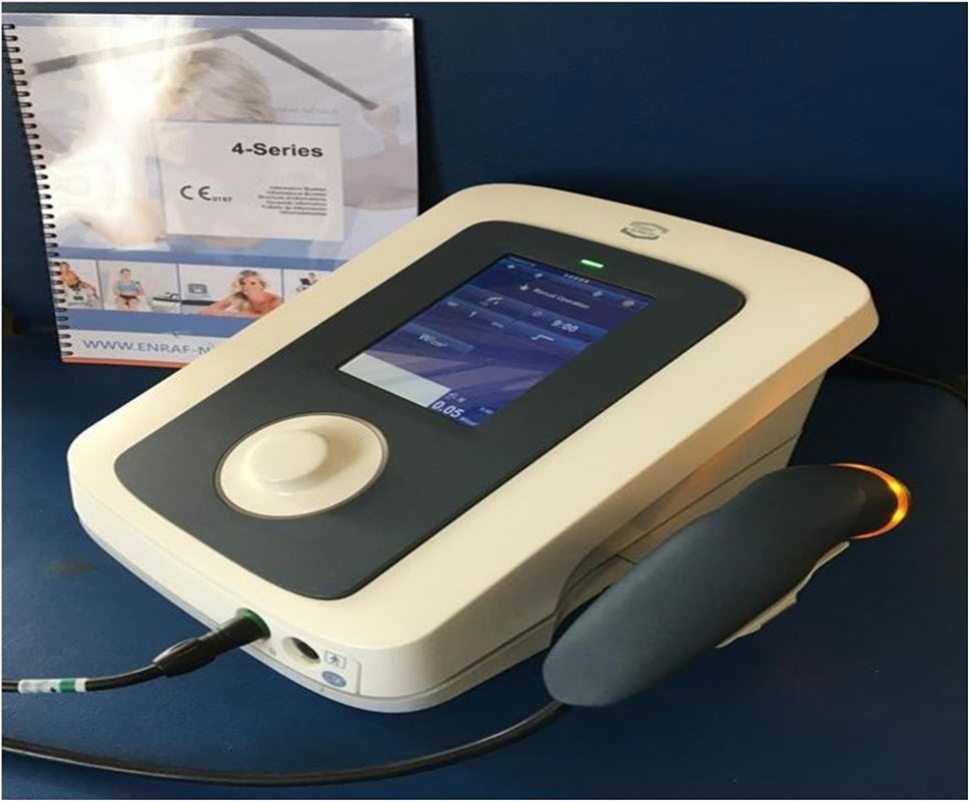
Sonopuls 490 ultrasound therapy device

Group B got continuous US waves by Sonopuls 490 ultrasound therapy device from Enraf-Nonius as shown in Fig. 3, frequency of 1 MHz and 1–1.5 W/cm^2^ power. US was performed bilaterally to cover the trapezius and sternocleidomastoid muscles 10 minutes each site for total 20-min session. The dosage was adjusted to the anatomical region of the neck. The treatment was applied by using circular movement with 5 cm^2^ —US head placed directly upon same points [12].

Evaluation methods are as follows:

1. The Cobb angle

**Fig. 4:**
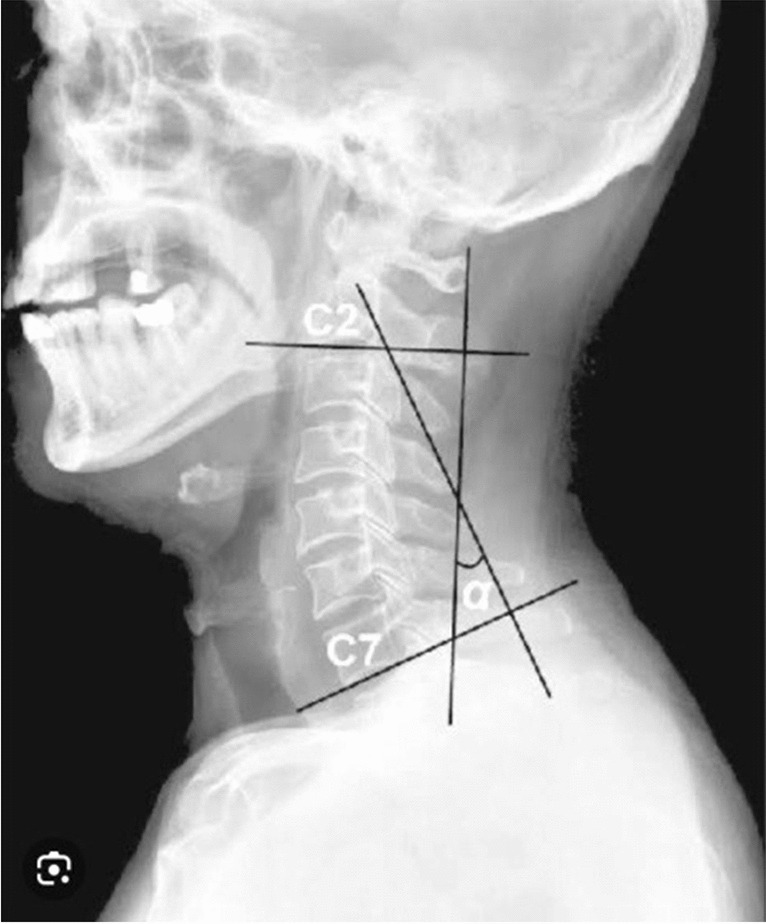
Cobb angle measurement

Cobb angle between C2 and C7 (Fig. 4) has been calculated using X-ray image. Drawing two parallel lines to the bottom endplates of C2 and C7, two lines that are perpendicular to each of these lines, and measuring the angle formed by the junction of the two orthogonal lines were used to determine this angle [13].

Patients were evaluated by X-ray measurement of Cobb angle, before and after treatment in all groups. X-rays were done to measure the improvement of cervical curve in all treatment groups; also, for its validity, we used Cobb angle to show the degree of improvement before and after treatment.

2. Visual analog scale (VAS)

VASs are well-known as reliable measurement methods for quantifying pain intensity [7]. It consists of a line usually 100 mm in length, with anchor descriptors such as (in the pain context) “no pain” and “worst pain imaginable.” The patient produces a mark that reflects his or her perception, and the distance from the left endpoint to the mark is measured in millimeters.

VASs were used before and after treatment scored from 0 which is no pain and 5 is the worst pain.

##### Sample size

Based on previous studies [14, 15], the mean VAS score in the laser group was decreased from 5.7 (4.8–7) pre-treatment to 0.5 (0.1–8) post-treatment; in continuous ultrasound, vas score was decreased from 5.9 (4.9–6.7) pre-treatment to 1.1 (0.4–7) post-treatment; in exercise group, it was decreased from 7.2 pre-treatment to 1.6 post-treatment. To detect the true difference between groups with a power of 80% and a level of significance of 5% and an effect size of 0.18, a minimum sample size of 45 participants will be needed (i.e., 15 participants) for each group, to compensate for non-parametric use, 25% will be added; therefore, a total sample size of 60 participants will be needed (i.e., 20 participants for each group). The sample size was calculated by G*Power (version 3.1.9.2; Germany).

##### Statistical methods

Version 28 of the Statistical Package for the Social Sciences (SPSS) was used for data coding and entry (IBM Corp., Armonk, NY, USA). Mean and standard deviation were used to represent quantitative data, while frequencies (number of cases) and relative frequencies (percentages) were used to summarize categorical data. Analyses of variance (ANOVA) with multiple comparisons post hoc tests were utilized to compare groups where the quantitative variables were normally distributed, while the non-parametric Kruskal-Wallis test and the Mann-Whitney test were employed when the quantitative variables were not regularly distributed. The paired *t* test was utilized to compare pre- and post-test values among the same group, whereas the non-parametric Wilcoxon signed-rank test was employed to compare non-normally distributed pre- and post-test values [16]. The chi-square [2] test was used to compare the two sets of categorical information. When the estimated frequency was less than 5, an exact test was used [17]. Statistical significance was assumed for *P*-values less than 0.05.

## Results

This study was designed as a prospective, randomized, controlled study. A sample of 60 children of both genders was recruited from pediatric laser clinic in National Institute of Laser Enhanced Sciences in Cairo University (NILES) and pediatric physiotherapy clinic at faculty of physical therapy, who were suffering from neck pain and X-ray shows straight cervical curve. They were randomly distributed into three groups equal in numbers; group A (20 patients received low-level laser therapy+ stretch and strength exercise three times per week for 4 weeks), group B (20 patients received therapeutic ultrasound therapy+ stretch and strength exercise, three times per week for 4 weeks), and group C (20 patients received stretch and strength exercise, three times per week for 4 weeks).

For groups (A), (B), and (C), the mean ± SD age was 15.26±1.28 years, was 16.25±1.16 years, and 14.85±1.60 years, with significant *P*-value < 0.001, < 0.001, and 0.006, respectively, as shown in Table 1. Also, there was no significant difference between all groups regarding the gender of patients (Table 2).

**Table 1:** The mean value, standard deviation, and *P*-value of age in the three treatment group

	Low-level laser therapy		Ultrasound therapy		Control group		*P-*value
	Mean	SD	Mean	SD	Mean	SD	
Age	15.26	1.28	16.25	1.16	14.85	1.60	0.006

**Table 2:** Count, percentage, and *P*-value of gender in each treatment group

		Low-level laser therapy		Ultrasound therapy		Control group		*P*-value
		Count	%	Count	%	Count	%	
Gender	Female	14	73.7%	12	60.0%	10	50.0%	0.315
	Male	5	26.3%	8	40.0%	10	50.0%	

The results were obtained from lateral cervical X-ray showing the cervical vertebrae curve shape and cervical Cobb angle before and after treatment, and the pain intensity were measured by visual analog scale VAS before and after treatment in each group.

**Fig. 5:**
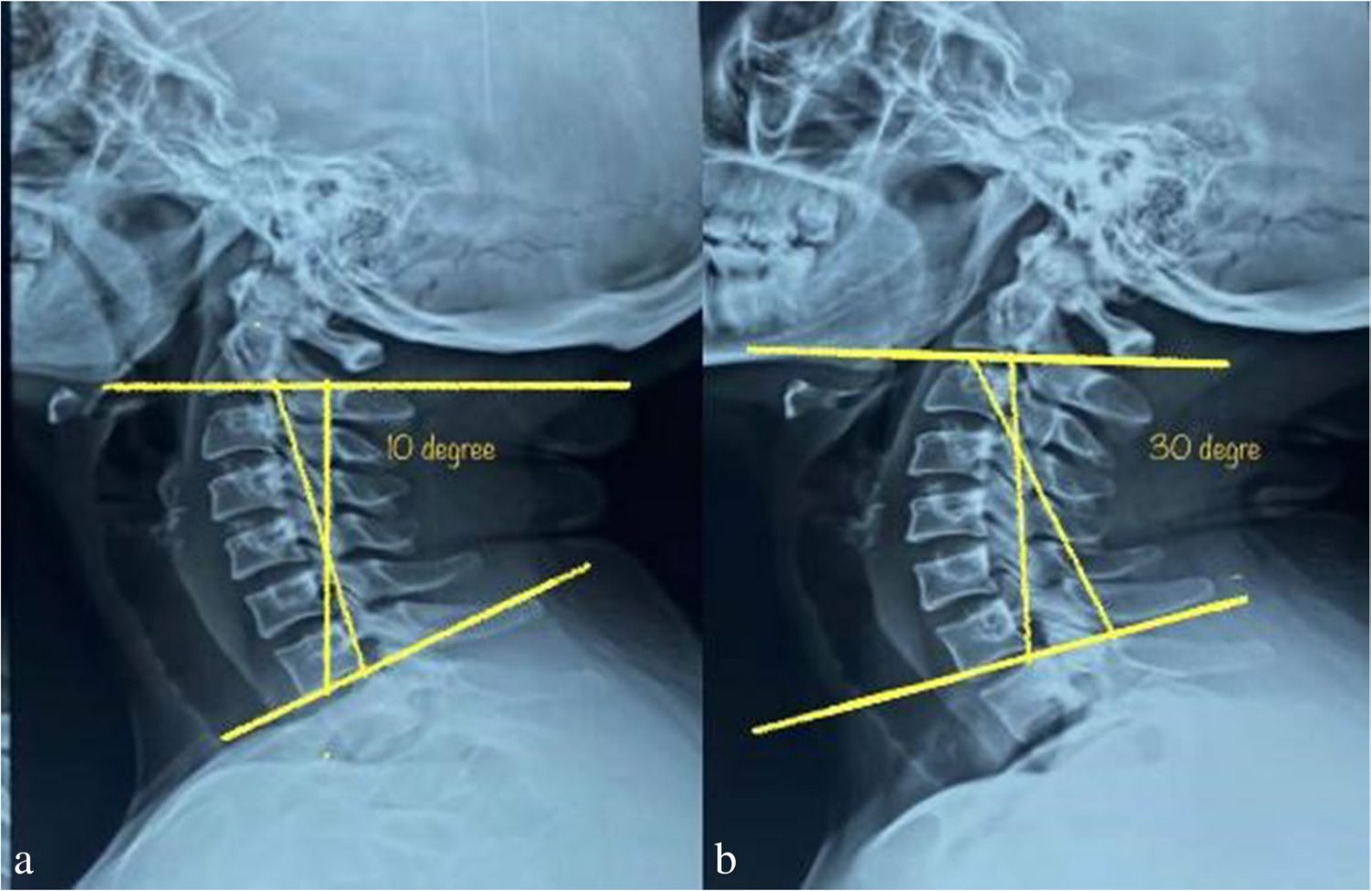
Patient X-ray before (**a**) and after (**b**) treatment with LLLT showing maximum improvement of cervical curve in group A

**Fig. 6:**
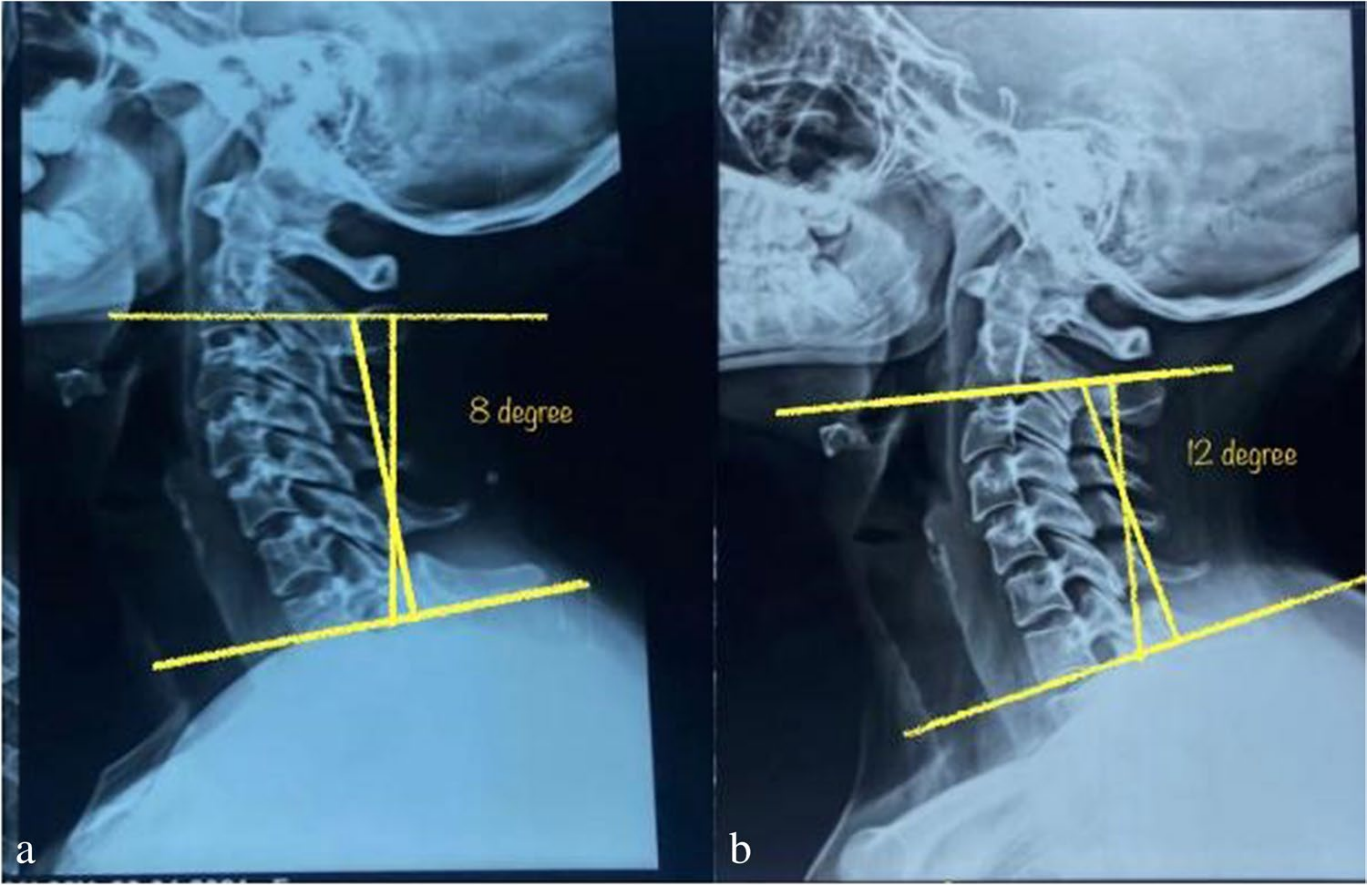
Patient X-ray before (**a**) and after (**b**) treatment showing minimal improvement of cervical curve in group C

In measuring the cervical curve correction of patients, we compared the three treatment group mean ± SD and *P*-value of Cobb angle before treatment; there was no significance with *P*-value 0.299. But after treatment, there was a significant improvement with *P*-value 0.002 more in laser group and US group, and least improvement was in the control group (Table 3 and Figs. 5 and 6(a, b)).

**Table 3:** Mean, standard deviation, and *P*-value of Cobb angle before and after treatment in low-level laser therapy, ultrasound therapy group, and diclofenac in gel form group

	Low-level laser therapy		Ultrasound therapy		Control group		*P*-value
	Mean	SD	Mean	SD	Mean	SD	
Cobb angle before	22.05	5.43	20.50	6.08	23.58	4.10	0.299
Cobb angle after	31.68	4.71	27.50	5.86	24.92	3.60	0.002
VAS before	4.05	0.78	4.30	0.57	4.33	0.49	0.381
VAS after	0.53	0.70	1.80	0.77	3.17	0.72	< 0.001

In measuring pain, we compared the three groups mean ± SD and *P*-value of visual analog scale before and after treatment. There was no significance with *P*-value 0.381. But after treatment, there was a significance with *P*-value <0.001 within laser group (Table 3).

## Discussion

The aim of the current study was to compare the effect of low-level laser, ultrasound, and exercise on pain that caused by straight neck deformity in school age children and teenagers who spent long time using electronic devices specially mobile phones (as the most used device in this age). There was a significant reduction in pain levels across all treatment groups by visual analog scale and improvement of the C shape of cervical vertebra by Cobb angle on patient X-ray, but the low-level laser therapy demonstrated the best results.

In the current study, we are not only measuring the improvement of neck pain by VAS like most of the studies, but also for our known knowledge; it was the first time to use Cobb angle measured by X-ray to evaluate the improvement of the cervical curvature after treatment of LLLT, US, and exercise, so we have to demonstrate the cause of straight or text neck syndrome; the results approve that the cervical flexion angle affects the muscle fatigue of right upper trapezius muscle and left upper trapezius muscle. Healthy cervical spine has a forward convex C curve [6]. That causes the anterior compartment muscles of the neck much less flexible, and it may overstretch additional muscles like the levator scapulae and sternocleidomastoid, limiting motion. In addition, it places an abnormal amount of strain on the ligament nuchae. Other abnormal curves of the spine, known as military back, can develop if the problem is not corrected [18].

Despite laser and ultrasound have good effects on both groups, there is a significant variation because absorption happens differently in various tissues [19]. Optimal tissue penetration is achieved due to the low absorption coefficient of laser radiation [20], but this absorption can either inhibit or promote cell and enzyme activity due to the production of histamine, serotonin, prostaglandin, and bradykinin [21].

There are few previous studies regarding LLLT effects on muscle hardness. In agreement of our study, Gür et al. reported that 904-nm LLLT (2 J/cm^2^ at each point) applied to the tender point of patients with fibromyalgia significantly decreased muscle spasms. Given that spasms are apt to occur due to muscle hardness, the red waveband and especially the near-infrared lasers which offer deeper penetration in biologic tissues could influence muscle hardness and attenuate it [22]. In fact, 810-nm LLLT used in the present study could decrease muscle spasm, so it shows improvement on cervical straightening shape measured by X-ray and Cobb angle. Recently, the high intensity laser was used in treatment of musculoskeletal spasm and was effective in reducing pain and improving function [23].

Another possibility regarding one of the most important effects underlying the mechanism of LLLT is the effect on the ATP-generating system. Karu et al. [24] reported that 632-nm LLLT increased the amount of ATP within irradiated muscle cells. In endurance training in human subjects, Vieira et al. reported that 808-nm LLLT showed a significant decremented of muscle fatigue and suggested that LLLT had positively affected the ATP synthesis process and/or lactate resolution [25]. Therefore, we could conclude that the wavelength-dependent LLLT effects in the present study were effective in improving muscle quality and so observing improvement of pain and cervical Cobb angle.

In disagreement of our study, a study in 2020 compared LLLT with conventional physical therapy and conventional physical therapy only in reducing pain levels, improving range of motion and reducing disability scores in patients having trigger points which is a point is an irritable spot within a taught band of skeletal muscle, ligament, or fascia in trapezius upper fibers. They indicated that patients having upper trapezius trigger points benefit from traditional physiotherapy, with or without low-level laser therapy. Similar to our findings, they discovered that the two together are more efficient than physiotherapy alone at reducing pain. The two approaches had comparable effects on cervical mobility [26].

For example, a 2017 study by Lippa et al. [27] found that a lack of cervical lordosis puts stress on the posterior longitudinal ligaments and elevates the load on the anterior disc, both of which can lead to pain. This is in line with our findings that cervical lordosis can be restored and kept stable with isometric training, which in turn facilitates range of motion at the cervical region.

Most recent studies have shown that ultrasound therapy is of poor quality. Latest evidence, however, has shown that ultrasound therapy can be utilized to deactivate trigger points associated with muscle pain syndrome. Two prior placebo-controlled investigations found that low-intensity US therapy improved pain pressure threshold and lowered sensitivity on the trigger point by producing transient effect on trigger points [28, 29].

In agreement of our study, Dundar et al. [30] evaluated the effect of ultrasound therapy on cervical myofascial pain syndrome; US was performed on trapezius muscle over 8 min (1.5 watt/cm^2^ dose, 1 MHz frequency, continuous mode US) (in a 3-week period). That shows significant improvement in visual analog scale before and after treatment after 15 sessions**,** while in our study, in US group, every patient took 20 min bilateral sides on neck muscles (1.5 watt/cm^2^ dose, 1 MHz frequency, continuous mode US) (in a 3 sessions per week for 4-week period) and shows significant improvement of visual analog scale, X-ray cervical curve, and Cobb angle pre- and post-treatment.

Dynamic neck exercises are a type of progressive resistance exercise that targets the neck as well as other body areas. Exercises that incorporate resistance, such as isometric, isokinetic, or isotonic movements, are considered strengthening exercises. It could involve using machines for strength training, using a thera-band, free weights, or doing low-intensity endurance exercises to develop muscular control [31].

In our study, patients received stretching and strengthening exercise which shows significant in visual analog scale and Cobb angle but was the weakest group in improvement compared with other group.

The method through which stabilizing and dynamic exercises have a therapeutic effect on patients with non-specific neck pain, according to Ylinen [32], is that strength or endurance activities work to strengthen the cervical muscles, which are prone to weakness with neck discomfort.

In a study evaluating the effects of 10 weeks of dynamic strength, endurance, and coordination exercises on pain and physical performance, Ahlgren et al. [33] found that strengthening exercises for the shoulders and upper extremities improve function while reducing pain caused by the trapezius muscles. However, the effects were gone by the follow-up at 8 months.

## Conclusion

Low-level laser therapy is the most effective method in treatment of straight neck syndrome and decreasing pain. It is safe noninvasive tool of treatment in children. Cobb angle measurement is an effective and subjective method for evaluation of neck deformity in children.

## References

[1] Domingues-Montanari S (2017). Clinical and psychological effects of excessive screen time on children. J Paediatr Child Health.

[2] Cochrane ME, Tshabalala MD, Hlatswayo NC, Modipana RM, Makibelo PP, Mashale EP, Pete LC (2019). The short-term effect of smartphone usage on the upper-back postures of university students. Cogent Eng.

[3] Kim S-Y, Koo S-J (2016). Effect of duration of smartphone use on muscle fatigue and pain caused by forward head posture in adults. J Phys Ther Sci.

[4] Park J, Kim K, Kim N, Choi I, Lee S, Tak S (2015). A comparison of cervical flexion, pain, and clinical depression in frequency of smart-phone use. Int J BioSci Biotechnol.

[5] Alsiwed KT, Alsarwani RM​, Alshaikh SA, Howaidi RA, Aljahdali A, Bassi MM, (2021). The prevalence of text neck syndrome and its association with smartphone use among medical students in Jeddah. Saudi Arabia. J Musculoskelet Surg Res.

[6] Hansraj KK (2014). Assessment of stresses in the cervical spine caused by posture and position of the head. Surg Technol Int.

[7] Dimitriadis Z, Strimpakos N, Kapreli E, Oldham J (2014). Validity of visual analog scales for assessing psychological states in patients with chronic neck pain. J Musculoskelet Pain.

[8] Mussttaf RA, Jenkins DF, Jha AN (2019). Assessing the impact of low level laser therapy (LLLT) on biological systems: a review. Int J Radiat Biol.

[9] Kolu E, Buyukavci R, Akturk S, Eren F, Ersoy Y (2018). Comparison of high-intensity laser therapy and combination of transcutaneous nerve stimulation and ultrasound treatment in patients with chronic lumbar radiculopathy: a randomized single-blind study. Pak J Med Sci.

[10] Mahamed A (2022). Efficacy of resisted exercise in straightened cervical lordosis: a case report: ABAB design.

[11] Klimo P, Rao G, Brockmeyer D (2007). Congenital anomalies of the cervical spine. Neurosurg. Clin N Am.

[12] Karkousha RN, Asar DAA, ElKeblawy MA, Kadah MA-E (2023). Acupoint focused ultrasound versus laserpuncture in chronic mechanical neck pain. J Popul Ther Clin Pharmacol.

[13] Kim S, Mo S, Moon W, Jun P, Kim C (2016). Effects of cervical kyphosis on recovery from dysphagia after stroke. Annals of Rehabilitation Medicine..

[14] Cunha A, Burke T, França F, Marques A (2009). Effect of global posture reeducation and of static stretching on pain, range of motion, and quality of life in women with chronic neck pain: a randomized clinical trial. Clinics (São Paulo, Brazil).

[15] Rubira AP, Rubira MC, Rubira LD, Comachio J, Magalhães MO, Marques AP (2019) Comparison of the effects of low-level laser and pulsed and continuous ultrasound on pain and physical disability in chronic non-specific low back pain: a randomized controlled clinical trial. Adv Rheumatol 59(1). 10.1186/s42358-019-0099-z 10.1186/s42358-019-0099-z 31847915

[16] Chan YH (2003). Biostatistics102: quantitative data – parametric & non-parametric tests. Singapore Med J.

[17] Chan YH (2003). Biostatistics 103: qualitative data –tests of independence. Singapore Med J.

[18] Sakthivel P, Singh CA, kumar D (2019). Military neck. Archives of Radiology.

[19] Watson T (2008). Ultrasound in contemporary physiotherapy practice. Ultrasonics.

[20] Kulekcioglu S, Sivrioglu K, Ozcan O, Parlak M (2003). Effectiveness of low-level laser therapy in temporomandibular disorder. Scand J Rheumatol.

[21] Bortoletto R (2004). Silva Nd, Zangaro R, Pacheco M, Da Matta R. Pacheco-Soares C. mitochondrial membrane potential after low-power laser irradiation. Lasers Med Sci.

[22] Gür A, Karakoc M, Nas K, Cevik R, Sarac J, Demir E (2002). Efficacy of low power laser therapy in fibromyalgia: a single-blind, placebo-controlled trial. Lasers Med Sci.

[23] Tuan SH, Sun SF, Huang WY, Chen GB, Li MH, Liou IH (2022). Effect of high intensity laser therapy in the treatment of acute atlantoaxial rotatory subluxation: a case report. J Back Musculoskelet Rehabil.

[24] Karu T, Pyatibrat L, Kalendo G (1995). Irradiation with He-Ne laser increases ATP level in cells cultivated in vitro. J Photochem Photobiol B Biol.

[25] de Vieira B, Hérickson W, Ferraresi C, de Perez A, Eduardo S, Baldissera V, Parizotto NA (2012). Effects of low-level laser therapy (808 nm) on isokinetic muscle performance of young women submitted to endurance training: a randomized controlled clinical trial. Lasers Med Sci.

[26] Waseem I, Tanveer F, Fatima A (2020). Can addition of low level laser therapy to conventional physical therapy be beneficial for management of pain and cervical range of motion in patients with trigger point of upper trapezius?. Anaesth Pain Intensive Care.

[27] Lippa L, Lippa L, Cacciola F (2017). Loss of cervical lordosis: what is the prognosis?. J Craniovertebr Junction Spine.

[28] Srbely JZ, Dickey JP, Lowerison M, Edwards AM, Nolet PS, Wong LL (2008). Stimulation of myofascial trigger points with ultrasound induces segmental antinociceptive effects: a randomized controlled study. Pain.

[29] Srbely JZ, Dickey JP (2007). Randomized controlled study of the antinociceptive effect of ultrasound on trigger point sensitivity: novel applications in myofascial therapy?. Clin Rehabil.

[30] Dundar U, Solak O, Samli F, Kavuncu V (2010). Effectiveness of ultrasound therapy in cervical myofascial pain syndrome: a double blind, placebo-controlled study. Turk J Rheumatol.

[31] Kaka B, Omoyemi O, Ogunlade S, Adeniyi A (2015). Effects of neck stabilization and dynamic exercises on pain, disability and fear avoidance beliefs in patients with non-specific neck pain; a randomized clinical trial. Arch Physiother Glob Res.

[32] Ylinen J, Takala EP, Nykanen M (2003). Active neck muscle training in the treatment of chronic neck pain in women: a randomized controlled trial. J Am Med Assoc.

[33] Ahlgren C, Waling K, Kadi F, Djupsjö Backa M, Thornell LE, Sundelin G (2001). Effects on physical performance and pain from three dynamic training programs for women with work-related trapezius myalgia. J Rehabil Med.

